# Network Analysis of the Participation and Activity Inventory for Children and Youth (PAI-CY) 7–12 Years with Visual Impairment

**DOI:** 10.1167/tvst.9.6.19

**Published:** 2020-05-19

**Authors:** Ellen B. M. Elsman, Carel F. W. Peeters, Ruth M. A. van Nispen, Ger H. M. B. van Rens

**Affiliations:** 1 Department of Ophthalmology, Amsterdam UMC, Vrije Universiteit Amsterdam, The Amsterdam Public Health Research Institute, Amsterdam, The Netherlands; 2 Department of Epidemiology & Biostatistics, Amsterdam UMC, Vrije Universiteit Amsterdam, The Amsterdam Public Health Research Institute, Amsterdam, The Netherlands; 3 Department of Ophthalmology, Elkerliek Hospital, Helmond, The Netherlands

**Keywords:** low vision, children, network analysis, questionnaire development, participation

## Abstract

**Purpose:**

Children with visual impairment often experience more difficulties regarding participation compared to sighted peers. The Participation and Activity Inventory for Children and Youth (PAI-CY) has recently been developed to assess their participation needs. A novel application in the field of questionnaires is the use of network analysis to explore interrelations between items in order to capture their complex interactions as a reflection of the overall construct of measurement. This study aimed to apply network modeling for the PAI-CY 7–12 from the perspectives of children and their parents.

**Methods:**

Children and their parents (*n* = 195) completed the 55-item PAI-CY via face-to-face interviews and a web-based survey, respectively. Internal consistency, test-retest reliability, and concordance between children and parents were investigated. Two networks were created, along with visualizations of shared and differential connections between children and parents.

**Results:**

Eight items were deleted. Network structures were dissimilar; for children, connections evolved around social contacts and school items, whereas for parents, mobility, leisure time, acceptance, self-reliance, and communication items prevailed. In the children's network, playing imaginary games, inviting a friend to play at home, and estimating the distance from others were most connected to other items.

**Conclusions:**

This study uniquely identifies connections between items of the PAI-CY 7–12, highlighting the different perspectives parents and children have on what defines participation, possibly implying that they perceive the relevance of various rehabilitation programs differently.

**Translational Relevance:**

Rehabilitation programs aimed at improving the most connected items might positively affect other items in the network, possibly improving children's participation.

## Introduction

Despite the low prevalence of childhood visual impairment (VI), its consequences should not be underestimated, as the cause of VI is mostly not curable and children have to live with the impairment for the rest of their lives. Studies have shown that children with VI often report to have worse quality of life,^1^^,^^2^ are less physically active,^3^^–^^6^ and spend more time alone than their sighted counterparts.^7^^,^^8^ Data from qualitative studies suggest that social relationships, participation, acceptance of the impairment, independence, and well-being are major themes influencing the lives of children with VI.^9^^,^^10^

Children with VI might benefit from low-vision rehabilitation care. Low-vision rehabilitation centers offer a wide variety of services, including diagnostics and visual functioning examinations, facilitating practical, pedagogical, and psychological support and providing low-vision aids and training in their use. Participation is often regarded as the most important outcome of low-vision rehabilitation for children, as successful participation has a positive influence on well-being and ultimately increases quality of life.^11^^–^^15^

In order to assess the participation needs of children with visual impairment, four age-specific versions of the Participation and Activity Inventory for Children and Youth (PAI-CY) were recently developed: 0 to 2 years, 3 to 6 years, 7 to 12 years, and 13 to 17 years. Its content was shaped by end-users (i.e., low-vision professionals, children with VI and their parents).^10^ The PAI-CY was then tested for feasibility, acceptability, clarity, and content relevance in a small-scale pilot study.^16^

An important next step has been the psychometric evaluation of the PAI-CY. Psychometric evaluation of measurement instruments often adopts either a formative approach, in which the measured construct is regarded as a common effect of its observables (i.e., the items), or a reflective approach, in which a construct is defined as a latent variable that determines observable characteristics of the construct.^17^ Using a reflective approach, psychometric properties of instruments can be investigated using classical test theory and item response theory (IRT). Many questionnaires in the field of low vision have been investigated using IRT models (as well as Rasch models, which is a special case of an IRT model^18^^,^^19^), including several age versions of the PAI-CY.^20^^,^^21^ However, for IRT analyses, extensive sample sizes are required (although Rasch models may require smaller sample sizes), which are challenging to achieve because low vision in children is rare. This is one of the reasons why it has been difficult to determine the underlying reflective structure, for example, of the PAI-CY 13–17 years version.

Recently, network modeling has been applied as a novel and alternative approach to facilitate the identification of connections between items as a reflection of the construct of measurement. A network model describes more general dependency structures between the items than a factor model does. Furthermore, network analysis allows for the evaluation of item communities (i.e., clusters of items that relate closely with each other) and can give insight into the connectedness or importance of items in the network (often referred to as “centrality”).^22^ However, it does not provide information about the structural validity of a questionnaire, which can only be established with more traditional approaches. Among others, network analysis has been used in research concerning mental disorders,^23^^,^^24^ personality,^25^^,^^26^ health-related quality of life,^17^ and empathy.^27^

The aim of the current study is to explore the interrelations between items as a reflection of the construct measured with the PAI-CY 7–12 from children's and their parents’ perspectives using a network approach. Prior to the network analysis, acceptability, comments, and suggestions for improvement of the PAI-CY 7–12 are assessed, along with some basic psychometric properties in order to select items for inclusion in the network analysis.

## Methods

The study protocol was approved by the Medical Ethical Committee of Amsterdam UMC, location VUmc, the Netherlands. The study adhered to the tenets of the Declaration of Helsinki. Written informed consent was obtained from parents of all participating children.

### Participants

Children aged 7 to 12 years registered at two low-vision rehabilitation centers in the Netherlands, and their parents/caretakers were invited to participate. Children with major cognitive impairment were excluded from the invitation by the low-vision rehabilitation centers. There was no restriction regarding visual performance, and children with any cause of VI were eligible to participate. Children and their parents had to have sufficient knowledge and understanding of the Dutch language.

### PAI-CY 7–12

The 55 items of the PAI-CY 7–12 were divided over nine domains: play (PL; 3 items), social contacts (SC; 6 items), mobility (MO; 4 items), leisure time (LT; 8 items), communication (CO; 12 items), school (SL; 11 items), self-reliance (SR; 5 items), acceptance/self-consciousness (AC; 5 items), and finances (FI; 1 item). Content and structuring of items were informed by qualitative research with end users.^10^

The PAI-CY 7–12 comprises a parallel child self-report and parent proxy-report format. The items of each of the formats are essentially identical, differing in developmentally appropriate language and use of first- and third-person tense, respectively. A 4-point ordinal response scale is used across both formats (1 = not difficult, 2 = slightly difficult, 3 = very difficult, 4 = impossible). The response option “not applicable” is treated as a missing value.

### Procedure

Participating parents completed the questionnaires through a web-based survey (a paper-and-pencil version was available on request), whereas children completed the questionnaires through face-to-face interviews in their own homes. For children, the questionnaires consisted of the PAI-CY 7–12 and a self-constructed evaluation form. Parents filled in questions about sociodemographic and clinical characteristics of their child and living situation, the PAI-CY 7–12, and a self-constructed evaluation form. Approximately 2 weeks after initial completion, participants were asked to complete a retest on the PAI-CY 7–12. Children were interviewed by the same interviewer. Ophthalmic information of children (visual acuity, visual field, and diagnosis) was retrieved from the patient files at the low-vision rehabilitation organizations. Missing values were complemented with self-reported data from parents (*n* = 16 for visual acuity and *n* = 33 for diagnosis). Visual acuity was classified in five levels based on the better-seeing eye, according to the criteria of the World Health Organization.^28^ A visual field ≤10 degrees was classified as blind; otherwise, only visual acuity was used for classification.

### Statistical Analyses

Descriptive statistics were used to analyze sociodemographic and clinical characteristics, as well as response frequencies to the PAI-CY 7–12 and to the evaluation forms. Comments and suggestions on the evaluation forms were comprehensively examined. Items with missing scores >40% in both the self-report format and proxy-report format were deleted from further analyses, whereas items with missing scores >20% were considered for deletion. Items with scores >70% in the first or last answer category (representing floor and ceiling effects) were also considered for deletion.

Internal consistency was investigated for the self-report format and the proxy-report format by calculating Spearman interitem correlations, item-total correlations, and Cronbach's alpha. Item pairs with interitem correlations >0.7 were considered for deletion, as were items with an item-total correlation <0.3.^29^

Concordance between parent and child scorings per adjoining item was examined using Kendall's coefficient of concordance (Kendall's *W*).^30^ This coefficient can be considered an agreement statistic. A correction for ties was used in the calculation of Kendall's *W*. Bootstrapping was used to produce 95% confidence intervals around *W*. The number of bootstrap samples used was 2000. A value >0.7 is generally considered to indicate adequate agreement.

Test-retest reliability was investigated at item level, using weighted kappa and percentage agreement.^29^ Kappa values >0.4 were considered moderate, >0.6 good, and >0.8 very good,^31^ whereas agreement of 60% to 74% was considered moderate, 75% to 89% good, and ≥90% excellent.^32^

A two-dimensional maximum likelihood factor analysis was applied to the mixture of polytomous items under the assumption that the items follow a graded structure. The correlation between the two latent traits was relatively low (0.367), and the factor structure was not representative of a parent versus child trait. Moreover, the many violations of local independence as well as the limited amount of variance explained by the model led us to conclude that the IRT/factor approach is not an adequate description of the data-generating mechanisms. Hence, we applied network modeling as a more general approach to investigate the item interrelations. It should be noted that network modeling is not indicative for the factor structure of the PAI-CY 7–12, as opposed to traditional factor analysis.

### Network Analyses

Network analyses were conducted to assess the interrelations between items of the PAI-CY 7–12. A network consists of two elements: nodes, which represent the items, and edges, representing connections between pairs of nodes.^33^ Network extraction was based on graphical modeling, in which the support of the precision matrix (the inverse of the covariance matrix) represents a conditional independence graph. In such a graph, an edge represents a substantive partial correlation. Hence, linkage in a conditional independence network means that the association between two connected items cannot be explained away by conditioning on the other items. Two networks were extracted, one for the self-report format and one for the proxy-report format, as the items in these subclasses may be differentially connected. Extraction started with the Spearman correlation matrix based on pairwise-complete observations. The inverse of this raw correlation matrix was (for each subclass) based on a ridge estimate.^34^ A value for the associated penalty parameter was determined by assessing the condition number of the ridge estimate along the penalty domain.^35^ The value was chosen such that the approximate loss in digits of accuracy did not exceed 2. Support determination of the estimated precision matrix was subsequently based on a local false discovery rate procedure. Only those edges were retained whose posterior probability of being present equaled or exceeded .75. The resulting networks were visualized by using the Fruchterman-Reingold algorithm,^36^ which tends to place highly connected nodes toward the center of the network and tries to minimize the number of crossing edges. The node coordinates of the self-report network serve as the reference coordinates for the proxy-report network. Node importance was assessed through simple centrality scores,^37^ especially degree centrality, which assesses the (structural) importance of a node by counting how many connections it has. It is indicative of the nodes that are central or influential in terms of the number of connections: more connections could imply deeper regulatory influence.^33^^,^^38^ From a statistical viewpoint, a central item shares most of its variance with all other items. From a conceptual viewpoint, in the case of questionnaire data, a response to a central item might influence the response to other items that share a connection. For instance, a high-score response to the most central item might indicate a high-score response to all items the central item is connected to (if the items are positively connected).

## Results

### Participants

The first questionnaire was completed by 195 parents and/or children. Table 1 presents sociodemographic and clinical characteristics. Complete data were obtained from 184 child-parent dyads. The retest was completed by 186 children and 174 parents.

**Table 1.** Sociodemographic and Clinical Characteristics of Participants

Characteristic	Value
Age, mean ± SD (range)	9.44 ± 1.58 (7–12)
Male sex, *n* (%)	113 (57.9)
Severity of VI,^28^ *n* (%)	
No VI: logMAR ≤0.3, *n* (%)	89 (45.6)
Mild VI: logMAR 0.31–0.52	37 (19.0)
Moderate VI: logMAR 0.53–1.00	51 (26.2)
Severe VI: logMAR 1.01–1.30	1 (0.5)
Blind: logMAR ≥1.31 or visual field ≤10 degrees	13 (6.7)
Unknown	4 (2.1)
Site of VI, *n* (%)	
Whole globe and anterior segment	7 (3.6)
Glaucoma: primary or secondary	2 (1.0)
Cornea (sclerocornea and corneal opacities)	3 (1.5)
Lens (cataract and aphakia)	14 (7.2)
Uvea	0 (0.0)
Retina	62 (31.8)
Optic nerve	9 (4.6)
Cerebral/visual pathways	29 (14.9)
Other (idiopathic nystagmus, high refractive error)	53 (27.2)
Unknown	16 (8.2)
Comorbidity, *n* (%)	91 (48.4)
Method of completion parent, *n* (%)	
Online	185 (98.4)
Paper-and-pencil	3 (1.6)
Parent who completed the questionnaire, *n* (%)	
Mother	144 (76.6)
Father	21 (11.2)
Mother and father together	19 (10.1)
Caretaker	4 (2.1)
Dutch nationality parent, *n* (%)	175 (93.1)
Education in years parent, mean ± SD (range)	12.56 ± 3.11 (0–16)
Financial situation parent, *n* (%)	
Usually enough money	84 (44.7)
Just enough money	48 (25.5)
Not enough money	11 (5.9)
No answer	45 (23.9)

### Evaluation

**Figure 1.**
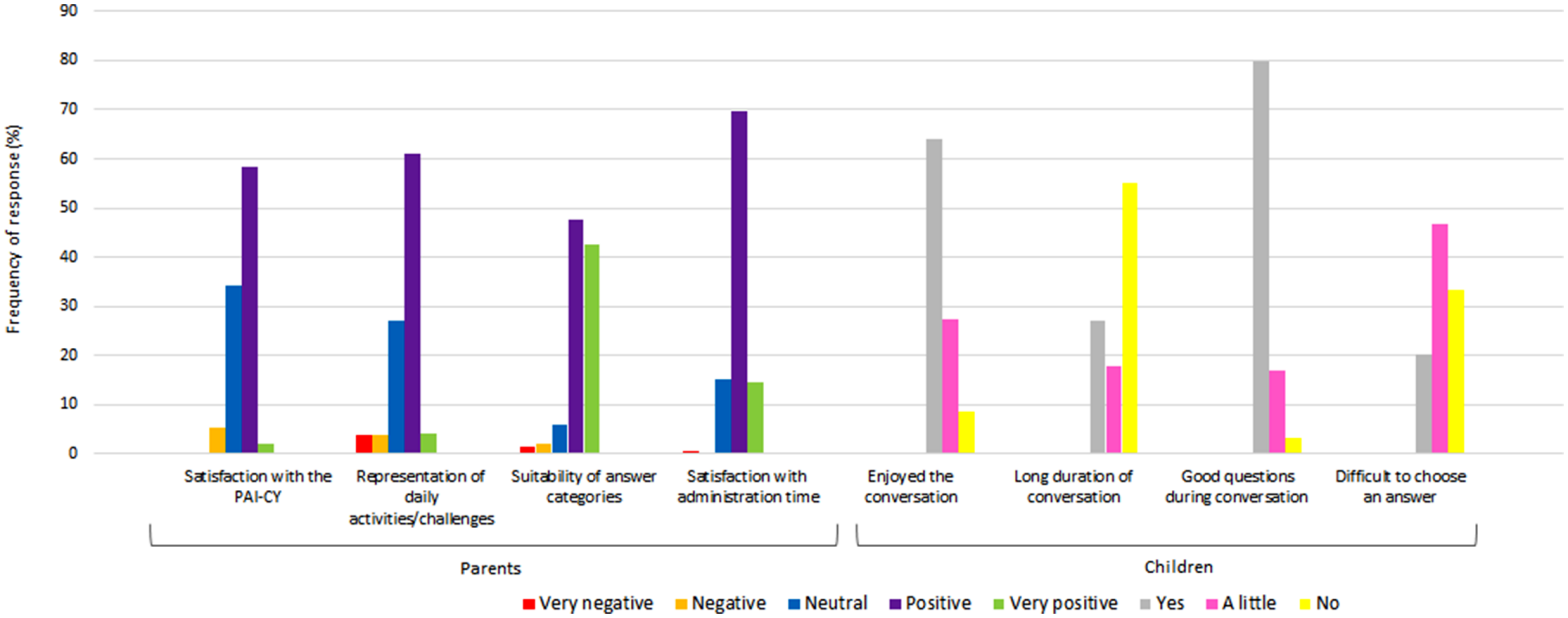
Evaluation of the PAI-CY 7–12 by children (*n* = 189) and parents (*n* = 185).

Over 90% of the parents were neutral to very positive regarding various aspects of the PAI-CY 7–12 (Fig. 1). Self-reported time to complete the PAI-CY 7–12 (including questions on sociodemographic and clinical characteristics) was 23.5 ± 15.2 (range 5–120, median 20) minutes. Children mostly enjoyed the conversation, and most children thought that the right questions were being asked. However, 27% of the children thought the interview took long, and 20% had difficulties selecting the right response option. This was reflected in the comments and suggestions by children and parents; several participants indicated the lacking response option “difficult.” Table 2 provides an overview of the comments and suggestions relevant to the PAI-CY 7–12 made by at least two participants and suggestions for solutions.

**Table 2.** Comments of Respondents (≥2) Relevant to the PAI-CY 7–12 and Suggested Solutions

Comments	Suggested Solutions
“I miss questions about the acceptation of the visual impairment”	*No adjustment* *—* *covered by items in the PAI-CY* *acceptance/self-consciousness* *domain* *and additional domain* *of* *parental experiences*
“Questions about making contact to other children are lacking”	*No adjustment* *—* *covered by items in the PAI-CY* *social contacts* *domain*
“Questions about finding your way in an unknown environment are lacking”	Adding item: MO: finding your way in an unknown environment
“I miss questions about the acceptance of parents”	*No adjustment* *—* *covered by items in additional domain parental experiences*
“Questions about the difficulty of darkness or too much light are lacking”	Adding item: MO: participating in traffic in the dark
“Questions about going to the toilet and bathing/showering are too easy/not age-appropriate”	Item SR4 (going to the toilet) deleted
“The textbox to give additional information could be larger”	Enlargement of the textbox
“A response option between slightly difficult and very difficult is missing”	Adding the response option “difficult”
“Questions CO9 and AC4 are similar”	*No adjustment* *—* *not supported by statistical analysis (* *i.e.,* *interitem correlations)*
“I would like to have more items regarding using the computer”	*No adjustment* *—* *sufficient items regarding gaming and using the computer*
“I miss items about playing at a playground or on playground equipment”	Adding item: PL: playing at a playground
“Items regarding playing independently are lacking, for example with Lego or dolls”	Adding item: PL: playing independently in your room with, for example, Lego or dolls
“Items about sleep are missing”	Adding item: AC: achieving sufficient sleep
“I want to have more items about playing outside”	*No adjustment* *—* *covered by items in the PAI-CY* *play and social contacts* *domains*

AC, acceptance/self-consciousness; MO, mobility; PL, play; SR, self-reliance.

### Item Analyses

One item (SL6, “Reading braille”) had a missing score of >40% in both the self-report format and the proxy-report format, resulting in deletion of this item. Eight items had missing scores of >20% in either self-report format or proxy-report format, of which four items had >20% missing scores in both formats (Table 3). Floor effects were found in 28 items in either the self-report format or proxy-report format, of which 12 items displayed floor effects in both formats. Infrequent endorsement of the fourth response category motivated collapsing this category with the third response category.

**Table 3.** Distribution of Responses over the Response Categories, Parameters for Test-Retest Reliability, and Concordance for the PAI-CY 7–12 Years

			Distribution of Responses				Test-Retest Reliability		
			over Response Categories,^*^ %				Parameters		
		Missing,					Agreement,	Weighted	
Domain and Item Content	Respondent	%	1	2	3	4	%	Kappa	Concordance
PL1: Playing imaginary games	Child	33.0	84.4	14.1	1.5	0.0	81.6	0.10	0.48
	Parent	8.5	77.9	17.4	3.5	1.2	90.1	0.73	
PL2: Playing a game with rules	Child	1.6	73.9	23.9	1.6	0.5	69.6	0.18	0.57
	Parent	1.1	53.2	37.1	9.1	0.5	73.3	0.64	
PL3: Keeping up with other children while playing	Child	1.0	50.8	36.0	13.2	0.0	60.9	0.49	0.57
	Parent	0.5	26.7	54.0	18.7	0.5	70.7	0.65	
SC1: Making contact with other children	Child	0.5	84.7	11.6	3.7	0.0	85.9	0.41	0.67
	Parent	0.0	67.0	27.1	5.9	0.0	80.5	0.71	
SC2: Doing activities with peers without visual impairment	Child	1.0	84.7	13.8	1.6	0.0	82.6	0.35	0.59
	Parent	0.0	60.6	33.5	5.9	0.0	75.0	0.60	
SC3: Playing outdoors with friends	Child	3.7	86.4	12.5	1.1	0.0	85.4	0.34	0.59
	Parent	2.7	59.6	30.6	7.7	2.2	72.8	0.60	
SC4: Playing at a friend's house	Child	6.3	87.2	11.2	1.1	0.6	85.2	0.39	0.62
	Parent	1.1	71.5	23.1	4.3	1.1	74.9	0.55	
SC5: Inviting a friend to play at your house	Child	7.9	92.6	4.5	2.3	0.6	85.9	0.24	0.55
	Parent	1.6	72.4	19.5	8.1	0.0	74.6	0.64	
SC6: Participating in group activities	Child	6.3	64.2	31.8	3.9	0.0	73.0	0.45	0.63
	Parent	1.6	35.7	42.7	19.5	2.2	66.1	0.66	
MO1: Cycling	Child	4.2	66.7	25.7	2.7	4.9	74.1	0.60	0.75
	Parent	1.6	53.0	30.8	11.4	4.9	77.4	0.76	
MO2: Doing activities with speed	Child	2.1	71.1	21.9	5.9	1.1	73.4	0.42	0.61
	Parent	3.2	36.8	37.9	19.8	5.5	63.9	0.64	
MO3: Participating in traffic independently	Child	10.5	68.4	19.9	4.7	7.0	69.7	0.53	0.66
	Parent	2.7	25.7	43.7	24.0	6.6	70.1	0.73	
MO4: Learning new routes	Child	4.2	66.7	25.7	7.7	0.0	69.1	0.40	0.60
	Parent	3.2	62.6	25.3	10.4	1.6	77.6	0.70	
LT1: Reading books	Child	6.8	64.0	25.8	7.9	2.2	74.9	0.65	0.71
	Parent	1.6	40.0	35.7	21.1	3.2	71.7	0.73	
LT2: Using social media	Child	33.5	85.0	13.4	0.8	0.8	83.6	0.48	0.57
	Parent	24.5	71.8	19.7	7.7	0.7	80.4	0.75	
LT3: Playing games on computer, tablet, phone	Child	4.2	84.7	13.7	1.1	0.5	86.9	0.46	0.65
	Parent	1.1	70.4	23.1	6.5	0.0	77.9	0.65	
LT4: Watching films/television	Child	3.7	82.6	14.7	2.7	0.0	83.8	0.37	0.62
	Parent	2.1	60.9	37.0	2.2	0.0	75.3	0.59	
LT5: Going to a club/association independently	Child	70.2	75.4	7.0	1.8	15.8	82.5	0.45	0.78
	Parent	28.2	33.3	33.3	17	16.3	73.3	0.77	
LT6: Participating at a club/association	Child	22.5	84.5	11.5	3.4	0.7	82.7	0.35	0.56
	Parent	11.7	47.6	39.2	12.0	1.2	70.3	0.59	
LT7: Making music	Child	59.7	67.5	29.9	2.6	0.0	72.6	0.35	0.48
	Parent	31.4	62.0	30.2	7.0	0.8	75.9	0.59	
LT8: Performing a hobby	Child	25.1	83.2	14.7	2.1	0.0	89.3	0.49	0.57
	Parent	31.9	61.7	32.8	4.7	0.8	76.8	0.57	
CO1: Expressing in words properly	Child	0.0	63.4	31.9	3.7	1.0	69.3	0.28	0.60
	Parent	0.5	65.8	28.9	5.3	0.0	78.4	0.66	
CO2: Asking questions	Child	2.1	78.1	20.9	1.1	0.0	80.7	0.43	0.58
	Parent	0.0	72.3	23.4	4.3	0.0	79.9	0.60	
CO3: Talking about feelings	Child	9.4	56.1	31.2	11.0	1.7	65.2	0.43	0.61
	Parent	0.5	40.4	45.7	13.3	0.5	71.1	0.66	
CO4: Sharing events	Child	4.2	84.7	12.0	2.7	0.5	84.3	0.41	0.63
	Parent	0.5	70.1	25.1	4.8	0.0	75.1	0.55	
CO5: Participating in a conversation	Child	2.1	81.3	15.0	3.7	0.0	77.3	0.45	0.61
	Parent	1.1	81.2	14.5	4.3	0.0	84.9	0.68	
CO6: Asking help from familiar people	Child	2.6	80.1	17.7	2.2	0.0	79.6	0.19	0.57
	Parent	1.1	69.4	25.8	4.8	0.0	76.0	0.53	
CO7: Asking help from unfamiliar people	Child	12.0	27.4	43.5	23.2	6.0	64.5	0.60	0.63
	Parent	3.7	31.5	45.9	21.0	1.7	70.7	0.67	
CO8: Indicating what can (not) be seen	Child	5.8	61.7	25.6	10.6	2.2	67.8	0.31	0.56
	Parent	2.1	41.3	41.3	16.8	0.5	63.7	0.53	
CO9: Estimating feelings of other children	Child	1.6	50.5	31.9	9.0	8.5	64.6	0.47	0.65
	Parent	1.6	45.9	35.7	17.3	1.1	74.6	0.77	
CO10: Estimating the distance to others	Child	1.0	72.0	20.1	6.9	1.1	72.7	0.40	0.56
	Parent	3.2	33.5	50.0	15.9	0.5	60.6	0.59	
CO11: Stating that you want to join in a group	Child	1.0	79.9	14.8	3.7	1.6	80.2	0.46	0.64
	Parent	0.5	49.7	36.9	13.4	0.0	69.9	0.65	
CO12: Dealing with bullying	Child	27.2	32.4	28.1	30.2	9.4	55.6	0.48	0.63
	Parent	17.6	24.5	47.1	25.8	2.6	69.7	0.63	
SL1: Finding the way in school	Child	0.0	93.7	5.2	1.0	0.0	91.9	0.36	0.64
	Parent	3.2	85.2	10.4	2.7	1.6	88.5	0.53	
SL2: Keeping overview in class	Child	0.0	85.3	12.6	2.1	0.0	83.8	0.35	0.55
	Parent	2.7	47.5	42.1	9.8	0.5	71.2	0.56	
SL3: Keeping up with classmates	Child	2.1	56.1	30.5	12.3	1.1	65.2	0.52	0.70
	Parent	2.7	37.7	42.6	17.5	2.2	71.6	0.74	
SL4: Reading the slide board or schoolboard	Child	5.2	63.0	21.0	9.9	6.1	70.2	0.52	0.63
	Parent	9.0	27.5	40.4	24.0	8.2	68.2	0.61	
SL5: Writing	Child	3.1	73.0	20.0	4.9	2.2	75.6	0.48	0.74
	Parent	5.9	33.9	35.0	28.2	2.8	68.1	0.69	
SL6: Reading braille	Child	91.1	82.4	17.6	0.0	0.0	–	–	–
	Parent	87.8	43.5	34.8	13	8.7	–	–	
SL7: Finding information	Child	28.8	69.9	24.3	4.4	1.5	75.9	0.47	0.53
	Parent	13.8	43.8	36.4	15.4	4.3	68.0	0.62	
SL8: Finding school stuff in the closet/drawer	Child	1.0	68.8	24.9	4.8	1.6	70.7	0.48	0.52
	Parent	2.7	47.5	38.8	13.1	0.5	65.1	0.52	
SL9: Cooperating with other children	Child	1.6	87.8	10.1	2.1	0.0	83.5	0.36	0.60
	Parent	0.0	64.9	31.4	3.7	0.0	79.1	0.63	
SL10: Participating in physical education	Child	0.5	83.2	14.7	2.1	0.0	83.0	0.45	0.51
	Parent	1.1	53.2	37.1	8.6	1.1	74.6	0.66	
SL11: Maintaining energy levels for fun activities	Child	0.0	68.1	23.0	8.9	0.0	74.7	0.54	0.65
	Parent	0.5	47.6	33.7	18.2	0.5	72.8	0.71	
SR1: Eating with fork and knife	Child	4.7	75.3	18.7	4.4	1.6	79.9	0.56	0.60
	Parent	2.7	55.2	36.6	7.1	1.1	75.7	0.64	
SR2: Making a sandwich	Child	6.3	76.0	16.8	5.6	1.7	83.5	0.68	0.71
	Parent	1.1	58.6	33.9	7.0	0.5	82.6	0.77	
SR3: Brushing your teeth independently	Child	1.0	85.7	12.2	2.1	0.0	85.1	0.43	0.58
	Parent	1.1	77.4	20.4	2.2	0.0	86.0	0.59	
SR4: Going to the toilet	Child	0.5	94.2	4.7	1.1	0.0	95.7	0.51	0.66
	Parent	0.5	93.6	5.9	0.5	0.0	93.6	0.57	
SR5: Bathing/showering independently	Child	4.2	85.2	13.1	1.1	0.5	88.3	0.60	0.63
	Parent	1.1	77.4	22.0	0.5	0.0	83.7	0.58	
AC1: Telling others about your visual impairment	Child	7.3	60.5	26.0	9.6	4.0	67.8	0.50	0.55
	Parent	9.0	43.9	39.2	15.2	1.8	68.2	0.62	
AC2: Dealing with incapability	Child	6.3	36.9	45.3	16.2	1.7	60.8	0.44	0.53
	Parent	3.7	28.2	47.5	23.8	0.6	63.6	0.58	
AC3: Dealing with making mistakes	Child	2.1	60.4	27.3	11.2	1.1	70.7	0.47	0.60
	Parent	0.0	27.7	43.1	29.3	0.0	59.9	0.53	
AC4: Empathizing with others	Child	1.6	61.2	29.8	6.4	2.7	65.6	0.40	0.52
	Parent	0.5	51.9	29.4	17.6	1.1	69.8	0.68	
AC5: Using visual aids	Child	23.6	90.4	9.6	0.0	0.0	87.8	0.32	0.57
	Parent	11.7	60.8	33.1	6.0	0.0	72.5	0.59	
FI1: Recognizing money	Child	1.0	70.9	21.7	6.9	0.5	85.2	0.67	0.69
	Parent	6.4	54.5	34.1	10.2	1.1	80.1	0.78	

AC, acceptance/self-consciousness; CO, communication; FI, finances; MO, mobility; LT, leisure time; PL, play; SC, social contacts; SL, school; SR, self-reliance.

* 1 = not difficult; 2 = slightly difficult; 3 = very difficult; 4 = impossible.

### Internal Consistency

High interitem correlations were found between item pairs SC2 (“Doing activities with peers without visual impairment”) and SC3 (“Playing outdoors with friends”), as well as MO3 (“Participating in traffic independently” and LT5 (“Going to a club/association independently”), while low item-total correlations were found for items SL1 (“Finding the way in school”), SR4 (“Going to the toilet”), and SR5 (“Bathing/showering independently”) in the proxy-report format. Cronbach's alpha was 0.96. In the self-report format, low item-total correlations were found for items PL1 (“Playing imaginary games”), SC4 (“Playing at a friend's house”), SC5 (“Inviting a friend to play at your house”), LT2 (“Using social media”), LT4 (“Watching films/television”), SL1 (“Finding the way in school”), SL2 (“Keeping overview in class”), and AC5 (“Using visual aids”). Due to too little complete cases, Cronbach's alpha could not be computed for the self-report format.

### Test-Retest Reliability

In the proxy-report format, item AC3 (“Dealing with making mistakes”) showed suboptimal test-retest agreement. Agreement was moderate for 56% of the items and good for 39% of the items. Two items showed excellent percentage agreement. Weighted kappa was moderate for 21 items and good for 33 items in the proxy-report format. In the self-report format, item CO12 (“Dealing with bullying”) showed suboptimal agreement, and two items showed excellent percentage agreement. Agreement was moderate for 43% of the items and good for 52% of the items. Nineteen items showed suboptimal kappa values. Weighted kappa was moderate for 32 items and good for two items in the self-report format (Table 3).

## Concordance

The concordance between children and parents was mostly low; only six items had concordance >0.7 (SL3, “Keeping up with classmates”; SR2, “Making a sandwich”; LT1, “Reading books”; SL5, “Writing”; MO1, “Cycling”; and LT5, “Going to a club/association independently”). Although three items from the school domain and two items from the leisure time domain had adequate concordance, no clear pattern could be observed; there were no items within the domains that scored notably better or worse than items in other domains.

From these results, it was decided to delete items SC2 (“Doing activities with peers without visual impairment”), LT5 (“Going to a club/association independently”), LT7 (“Making music”), CO2 (“Asking questions”), CO5 (“Participating in a conversation”), SL1 (“Finding the way in school”), and SR4 (“Going to the toilet”). The remaining 47 items were included in the subsequent analyses.

### Network Analysis

**Figure 2.**
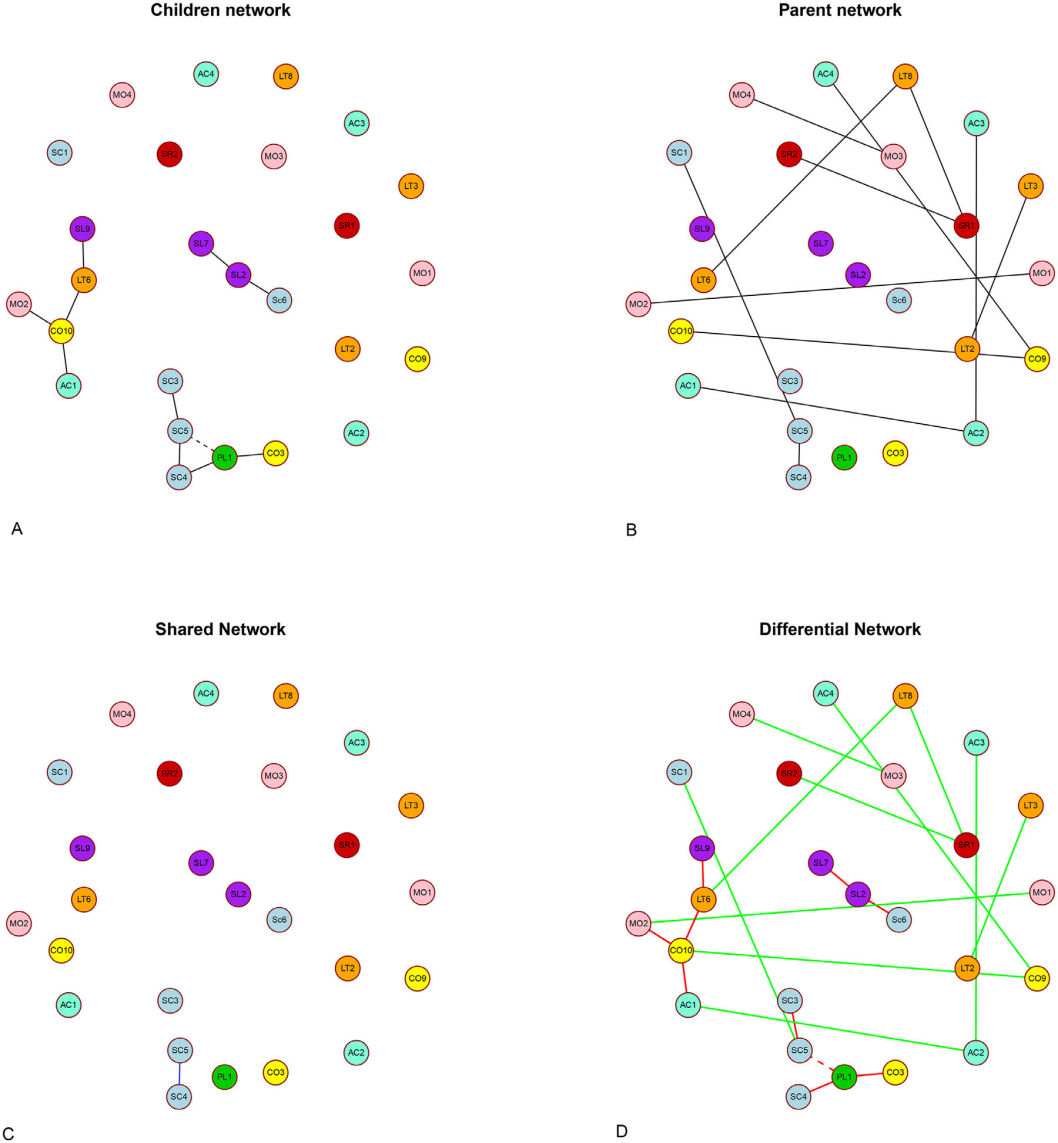
Networks visualized with the Fruchter-Reingold algorithm.^36^ (A) Network from children's perspective. (B) Network from parent's perspective. (C) Network consisting of edges that are shared between children and parent. (D) Network consisting of edges that are unique for both groups separately with *red edges* representing connections in children only, and *green edges* represent connections in parents only. *Solid edges* represent positive partial correlations, and *dashed edges* represent negative partial correlations. The nodes have different colors for every domain in the PAI-CY. *Blue*, social contacts; *green*, play; *purple*, school; *pink*, mobility; *red*, self-reliance; *yellow*, communication; *orange*, leisure time; and *turquoise*, acceptance.

Figures 2A and 2B show the networks of the children and parents, respectively. Edges between nodes within a network correspond to partial correlations between items, controlling for all other items. Positive connections are denoted by solid lines, whereas negative connections are denoted by dashed lines. Each node corresponds to a single item and has a color corresponding to its domain, as given in Table 3. Only those items that form connections are displayed in Figure 2 (i.e., 26 items, 55.3%). Node placement in the networks is based on the Fruchter-Reingold algorithm, which places more strongly connected nodes closer together.^36^

The results show that the network of children evolves around items in the social contacts and school domains. For parents, networks evolve around items in the social contacts domain as well, as around items in the mobility, leisure time, acceptance, self-reliance, and communication domains. All items are positively connected, except for the connection between items SC5 (“Inviting a friend to play at your house”) and PL1 (“Playing imaginary games”) in the children network.

Figure 2C shows the connections that are shared between the children and parent network, while Figure 2D shows the connections that are unique to either the children or the parent network. Only one connection (SC4, “Playing at a friend's house” and SC5, “Inviting a friend to play at your house”) is shared between the children and parent network, and most connections are unique to either the parent or children network.

In the children network, the items PL1 (“Playing imaginary games”), SC5 (“Inviting a friend to play at your house”), and CO10 (“Estimating the distance to others”) are the most central items, each having three connections to other items. SC5 (“Inviting a friend to play at your house”) is also the most central item in the parent network, as are items LT8 (“Performing a hobby”), CO9 (“Estimating feelings of other children”), SR1 (“Eating with fork and knife”), and AC2 (“Dealing with incapability”), all having two connections to other items.

## Discussion

The exploratory network analysis presented in this study is, to the best of our knowledge, the first applied in the field of low vision, highlighting how the interrelations of items reflect the construct of measurement of the PAI-CY 7–12. But first, some basic psychometric properties were assessed and found appropriate. Moreover, from the evaluation forms, it could be concluded that children and parents were generally satisfied with various aspects of the PAI-CY 7–12. Initial item deletion was done conservatively to preserve content validity, and only those items that were thought to be not relevant for the target population or showed similarity and overlap with other items were deleted.

Internal consistency for the proxy-report format was acceptable, and after item deletion, only two items showed low item-total correlations. Cronbach's alpha was high, which is probably caused by the high number of items.^29^ Cronbach's alpha of the final item set was slightly lower (i.e., 0.94, data not shown) but still at the high end, indicating possible item redundancy. In the self-report format, a larger number of items showed low item-total correlations, which might suggest suboptimal internal consistency among the items. Cronbach's alpha for the self-report format could not be computed due to too little complete cases but was expected to be high as well (of the final set of 47 items, Cronbach's alpha was 0.87 based on 10 complete cases).^29^ Test-retest reliability in the proxy-report format was satisfactory, but many items in the self-report format showed suboptimal kappa values while having adequate agreement. This might be due to symmetrically imbalanced contingency tables, known as the kappa statistic paradox.^39^^–^^41^

Concordance between children and parents was limited, suggesting that they perceive the impact of VI on participation differently, which is similar to previous studies.^42^^–^^44^ In over 75% of the dyads, the parents scored lower than their child, reflecting more disability, while the average score was also lower as assessed by parents than compared to children, which may indicate that parents underestimate their child's ability or that children overestimate themselves. It is known that children and parents differ in response styles. In contrast to parents, children tend to select more extreme scores, rate an item prior to provide a justification, and base their response on a single example.^45^ The different modes of administration between parents and children may have partly explained the lack of agreement. Children completed the questionnaires through face-to-face interviews, a method known to cause greater social desirability, yes-saying bias, and less willingness to disclose sensitive information.^46^ Besides methodological differences, discordance may arise because parents might already be focusing on future life demands and oversee the bigger picture, whereas children might focus on their current situation, without making comparisons to peers. This also became evident in the two networks that were created. The network of children shows that connections between items mainly evolved around items in the social contact and school domains. In the network of parents, connections between items evolved around items in the social contacts domain as well but also in the mobility, self-reliance, and acceptance domains. The prevailing domains in the network of children seemed to be more related to their current situation, whereas prevailing domains in the network of parents appeared to be related to future life demands. The differences between the networks of children and parents confirm the results of the concordance analyses and show that children and parents have a different view on what defines participation of children with VI aged 7 to 12 years. This also resonates with the results of the qualitative study on which the content of the PAI-CY 7–12 was based, in which parents focused on general development and increased awareness of the impairment, whereas children focused on social isolation and feeling dependent.^10^ The differences between the networks of children and parents might also imply that outcomes of rehabilitation programs might be perceived as different for children and their parents.

The data failed the core assumptions for IRT modeling (i.e., the unidimensionality and local independence assumptions).^47^ Hence, the factor approach does not give an adequate description of the data-generating mechanism, and thus we opted to find and describe more general network structures through network modeling. However, network modeling does not provide evidence for structural validity, as opposed to the more traditional approaches such as factor analysis. As such, we cannot draw firm conclusions about the psychometric properties of the PAI-CY 7–12 besides the results from the evaluation of some of the basic psychometric properties.

The networks created in this study, however, may inform directions for future research and practice. Centrality analysis showed that in the network of children, the items PL1 (“Playing imaginary games”), SC5 (“Inviting a friend to play at your house”), and CO10 (“Estimating the distance to others”) are most connected to other items and reflect the construct of participation from their perspectives. These findings suggest that when rehabilitation programs are provided to improve these activities, the other items in this network structure might also be positively affected. This is also the case for the items with high degree centrality in the network of parents, which were SC5 (“Inviting a friend to play at your house” as in the children's network) but also LT8 (“Performing a hobby”), CO9 (“Estimating feelings of other children”), SR1 (“Eating with fork and knife”), and AC2 “Dealing with incapability”). However, these items had fewer connections, thereby having less effect on the other items within the network. The cross-sectional nature of the data prevents making conclusions about causality (i.e., whether the most central items activate other items, are activated by other items, or both).

In conclusion, this study showed that most children and parents were satisfied with the PAI-CY 7–12, and internal consistency and test-retest reliability were acceptable. In addition, this study uniquely identified connections between items, highlighting different interpretations of the construct of participation between children and their parents. Future studies should try to obtain larger sample sizes in order to draw conclusions about differences in networks between various subpopulations, for example, with respect to severity or onset of VI. Larger samples might also enable more advanced analyses to inform further item deletion, thereby lowering the respondent burden. The network analysis allowed a better understanding of the construct of participation from children aged 7 to 12 years and their parents’ perspectives. This might also provide opportunities to focus on interventions that may improve participation levels of children with VI.
